# Current management of surgical neonates: is it optimal or do we need to improve? A national survey of the Italian Society of Neonatology

**DOI:** 10.1007/s00383-024-05680-6

**Published:** 2024-04-16

**Authors:** Simonetta Costa, Irma Capolupo, Luca Bonadies, Michele Quercia, Maria Pasqua Betta, Sara Gombos, Costanza Tognon, Giacomo Cavallaro, Stefania Sgrò, Roberta Pastorino, Denise Pires Marafon, Andrea Dotta, Giovanni Vento, on behalf of the Surgical Newborn Study Group of the Italian Society of Neonatology

**Affiliations:** 1 Department of Woman and Child Health and Public Health, Fondazione Policlinico Universitario Agostino Gemelli IRCCS, Rome, Italy; 2 Medical and Surgical Department of Fetus-Newborn-Infant, “Bambino Gesù” Children’s Hospital IRCCS, Rome, Italy; 3 Neonatal Intensive Care Unit, Department of Woman’s and Child’s Health, University of Padova, Padua, Italy; 4 Department of Biomedical Science and Human Oncology, Neonatology and Neonatal Intensive Care Section, University of Bari Aldo Moro, Policlinico Hospital, Bari, Italy; 5 Neonatal Intensive Care Unit, Azienda Ospedaliero-Universitaria Policlinico Rodolico San Marco, Catania, Italy; 6 Unit of Pediatrics, Santobono-Pausillipon Hospital, Naples, Italy; 7 Anesthesiology Pediatric Unit, Department of Women’s and Children’s Health, Padua University Hospital, Padua, Italy; 8 Neonatal Intensive Care Unit, Fondazione IRCCS Ca’ Granda Ospedale Maggiore Policlinico, Milan, Italy; 9 Sezione di Igiene, Istituto di Sanità Pubblica, Università Cattolica del Sacro Cuore, Rome, Italy; 10 Catholic University of Sacred Heart, Rome, Italy

**Keywords:** Neonatal surgery, Neonatal surgical management, Perioperative management, Bedside surgery, National survey, Italian Society of Neonatology

## Abstract

**Purpose:**

Few guidelines exist for the perioperative management (PM) of neonates with surgical conditions (SC). This study examined the current neonatal PM in Italy**.**

**Methods:**

We invited 51 neonatal intensive care units with pediatric surgery in their institution to participate in a web-based survey. The themes included (1) the involvement of the neonatologist during the PM; (2) the spread of bedside surgery (BS); (3) the critical issues concerning the neonatal PM in operating rooms (OR) and the actions aimed at improving the PM.

**Results:**

Response rate was 82.4%. The neonatologist is involved during the intraoperative management in 42.9% of the responding centers (RC) and only when the surgery is performed at the patient’s bedside in 50.0% of RCs. BS is reserved for extremely preterm (62.5%) or clinically unstable (57.5%) infants, and the main barrier to its implementation is the surgical-anesthesiology team's preference to perform surgery in a standard OR (77.5%). Care protocols for specific SC are available only in 42.9% of RCs.

**Conclusion:**

Some critical issues emerged from this survey: the neonatologist involvement in PM, the spread of BS, and the availability of specific care protocols need to be implemented to optimize the care of this fragile category of patients.

**Supplementary Information:**

The online version contains supplementary material available at 10.1007/s00383-024-05680-6.

## Introduction

Congenital anomalies requiring surgery significantly impact public health, both in terms of the quality of life of patients and their families and in terms of medical and social costs. They are associated with preterm delivery and low birthweight [1] and contribute to fetal and infant mortality, representing the fourth leading cause of death in children younger than 5 years in 2019, accounting for 9.4% of global death [2].

Prematurity is a condition at high risk for morbidities related to preterm birth requiring surgery, such as necrotizing enterocolitis (NEC) [3], hemodynamically significant patent ductus arteriosus (hsPDA) [4] unresponsive to medical therapy, post-hemorrhagic hydrocephalus as a consequence of intraventricular hemorrhage (IVH) [5], and severe forms of retinopathy of prematurity (ROP) [6].

Despite the magnitude of the problem, few guidelines exist for the perioperative management (PM) of newborn infants with specific surgical conditions (SC) [7], and both term and preterm neonates experience variable perioperative care, undergoing high rates of complications [8, 9]

As a preparatory step towards the standardization and optimization of the PM of newborn infants with SC, the Surgical Newborn Study Group (SNSG) of the Italian Society of Neonatology (SIN) conducted an official survey to investigate different aspects of the PM of term and preterm neonates in Italy.

## Methods

### Study design and participants

A prospective cross-sectional study was conducted via a web-based survey to assess the current PM of neonates with SC across Italian neonatal intensive care units (NICUs).

The heads of the 51 Italian NICUs with pediatric surgery in their institution were invited to respond to the survey. The 51 NICUs are evenly distributed across the country's 20 regions. These regions are classified into four main areas: Northern Italy, Central Italy, Southern Italy, and the Islands. Northern Italy comprises eight regions: Liguria, Piemonte, Valle D'Aosta, Lombardia, Emilia-Romagna, Trentino Alto-Adige, Veneto, and Friuli Venezia Giulia. Central Italy comprises four regions: Lazio, Marche, Toscana, and Umbria. Southern Italy encompasses six regions: Abruzzo, Basilicata, Calabria, Campania, Molise, and Puglia. The two main islands of Italy are Sicily and Sardinia.

The purpose of the investigation was to describe the landscape of PM of term and preterm neonates with SC in Italy, and the themes included: 1) the involvement of the neonatologist during the PM of the neonate and the kind of collaboration between the neonatologist and the anesthesiologist; 2) the spread of the practice of bedside surgery (BS), its characterization and the problems encountered in Italy for its implementation; 3) the critical issues related to the management of newborns in operating rooms (OR) and the actions aimed at improving the management of surgical neonates, including the presence of dedicated neonatal anesthesiologist, the use of care protocols for specific SC, and being a part of scientific study groups.

### Survey development

A survey of 12 questions was developed using a modified Delphi method [10]. Six authors (LB, MQ, MPB, SG, CT, GC), who are experts on the topic, commented on a previously prepared questionnaire (SC, IC). Consensus on a final version of the survey was reached after three rounds in which the experts proposed appropriate modifications to the questionnaire. The Commission for national and international fact-finding Investigations of the SIN finally approved the survey.

The survey was provided in Italian through the Survey Monkey online platform (https://www.surveymonkey.com). The survey was distributed through SIN.

The first sending was preceded by a cover letter reporting the purpose of the survey and the time required (approximately 15 min) to complete it. The survey remained open from May 1, 2023, to August 31, 2023. The first email was sent in May 2023, and three additional reminders were sent through August 2023. Those who did not respond were ultimately contacted via a personalized email and/or by telephone. No financial reward was offered for responding to the survey; the response to the survey implied the participant's informed consent.

The survey included several types of questions with different response options: dichotomous, multiple-choice, and open-ended response options. The survey questionnaire can be found in the Supplementary File.

The survey results were reported according to the checklist for reporting results of internet e-surveys (CHERRIES) [11].

### Statistical analysis

All responses were checked for duplicates from the same center. Only responses from unique Italian centers were included in the analysis. In case of more than one response per NICU, only the first response was included in the analysis. Any responses that were not clear or complete were excluded from the analysis.

Responses were tabulated, and results were reported as a percentage of the respondents. Categorical data were expressed as numbers and percentages and compared with the Chi-square or Fisher’s exact test, as appropriate. Data were analyzed with Stata software version 16 (StataCorp LP. College Station. TX, USA).

## Results

The overall survey response rate was 82.4%: 42 responding centers (RC) out of 51.

The geographical distribution of the RC was Northern Italy 18/42 (42.8%), 11/42 Central Italy (26.2%), Southern Italy and islands13/42 (31.0%).

### Involvement of the neonatologist during the neonatal perioperative management

The neonatologist was always involved during the intraoperative management in 42.9% of the RC (Table 1). In comparison, in 50.0% of the centers, the neonatologist was engaged only if the surgery was performed at the patient's bedside. In 7.1% of RC, the neonatologist was never involved in managing the critical neonatal issues during the surgery.

**Table 1:** Involvement of the neonatologist during the neonatal perioperative management

In your NICU, is the neonatologist involved in the intra-operative management of newborn infants with surgical conditions?	*N* = 42
Yes, always	18 (42.9)
No	3 (7.1)
The neonatologist is involved only if the surgery is performed at the bedside in the NICU	21 (50.0)
What is the collaboration between neonatologists and anesthesiologists regarding the management of infants with surgical pathology? (more than one answer is possible)	*N* = 42
All peri- and intraoperative management is referred to the anesthesiologist	3 (7.1)
Neonatologist and anesthesiologist share all critical issues before surgery	36 (85.7)
The infant is intubated by the neonatologist and then referred to the anesthesiologist	24 (57.1)
The neonatologist is involved in the management of ventilation during surgery only in case of high-frequency oscillatory ventilation	8 (19.0)
The neonatologist is involved in the management of ventilation during surgery regardless of the ventilation modality	15 (35.7)
The neonatologist is involved in fluid management during surgery	11 (26.2)
The neonatologist is involved in the pharmacological management of anesthesia	7 (16.7)
Neonatologist and anesthesiologist share antibiotic prophylaxis	20 (47.6)
Neonatologist and anesthesiologist share post-operative pain management	19 (45.2)

In most RC (85.7%), neonatologists and anesthesiologists shared all critical issues before surgery. In more than half of the centers (57.1%), the infant was intubated by the neonatologist and then referred to the anesthesiologist; in almost half of the cases (47.6%), the neonatologist and anesthesiologist shared antibiotic prophylaxis. During the intraoperative phase, the neonatologist was involved in the management of ventilation regardless of the ventilation modality in 35.7% of RC and only in the case of high-frequency oscillatory ventilation in 19.0% of RC; for the fluid management and the pharmacological management of anesthesia, the neonatologist was involved in 26.2 and 16.7% of cases, respectively. During the postoperative phase, neonatologists and anesthesiologists shared pain management in 45.2% of RC. Only in 7% of RC, all peri- and intraoperative management was referred to the anesthesiologist.

### Characterization of bedside surgery in Italy

Bedside surgery was performed in 95.2% of RC (Table 2). Among the 40 centers where BS was performed, BS was practiced for less than 5 years in 22.5%, 5–10 years in 12.5%, and more than 10 years in 65.0% of centers. BS practice facilitated collaboration between neonatologists and anesthesiologists in 85.0% of RC.

**Table 2:** Characterization of Bedside Surgery in Italy

Is bedside surgery performed in your NICU?	*N* = 42
Yes	40/42 (95.2)
No	2/42 (4.8)
How long has BS been practiced in your NICU?	*N* = 40
Less than 5 years	9/40 (22.5)
5–10 years	5/40 (12.5)
More than 10 years	26/40 (65.0)
Does BS facilitate collaboration between neonatologists and anesthesiologists?	*N* = 40
Yes	34/40 (85.0)
No	6/40 (15.0)
What are the criteria for BS in your center? (more than one answer is possible)	*N* = 40
All newborn infants with surgical pathology	1/40 (2.5)
Gestational age < 28 weeks and/or birthweight < 1000 g	25/40 (62.5)
Infants with clinical instability, regardless of gestational age and birth weight	23/40 (57.5)
Depends on ventilatory needs (e.g., neonates on HFOV or requiring inhaled nitric oxide)	18/40 (45.0)
Infants on extracorporeal oxygenation	5/40 (12.5)
Other	4/40 (10.0)
Which surgical pathology is preferentially managed at the bedside in your center? (more than one answer is possible)	*N* = 40
Abdominal surgery (NEC, SIP, abdominal wall defects)	27/40 (67.5)
Thoracic surgery (CDH, CPAM)	16/40 (40.0)
Heart surgery (PDA ligation)	33/40 (82.5)
Placement of drainage (pulmonary, abdominal, bladder)	34/40 (85.0)
Other	4/40 (10.0)
What are the biggest challenges to bedside surgery in your center? (up to 3 options)	*N* = 40
Structural critical issues (e.g., absence of adequate physical space)	19/40 (47.5)
No adequate standards for protection against infectious risk	9/40 (22.5)
Shortage of personnel to ensure an adequate surgical team	5/40 (12.5)
Preference of the surgical-anesthesiology team to perform surgery in standard OR	31/40 (77.5)
Administrative problems	2/40 (5.0)
Medico-legal doubts	4/40 (10.0)

*BS* bedside surgery, *CDH* congenital diaphragmatic hernia, *CPAM* congenital pulmonary airway malformations, *HFOV* high-frequency oscillatory ventilation, *NEC* necrotizing enterocolitis, *NICU* neonatal intensive care unit, *OR* operating room, *PDA* patent ductus arteriosus, *SIP* spontaneous intestinal perforation

Gestational age < 28 weeks and/or birthweight < 1000 g were the criteria to undergo BS in 62.5% of centers, whereas clinical instability, regardless of gestational age and birthweight, was the selection criterion for 57.5% of centers. Specific ventilatory needs such as high-frequency oscillatory ventilation or nitric oxide inhalation (45.0%) and extracorporeal oxygenation (12.5%) were the other most-reported criteria for BS. Only in 2.5% of centers with BS all newborn infants with surgical pathology were operated at the bedside.

Drainage placements (85.0%) and patent ductus arteriosus ligation (82.0%) were the surgical procedures most frequently performed at the bedside. Abdominal surgery (67.5%) and thoracic surgery (40.0%) were the other procedures considered for BS.

The preference of the surgical-anesthesiology team to perform surgery in standard OR was the major obstacle centers encountered for implementing BS (77.5%). In contrast, structural issues such as inadequate physical space in the NICU were located in 47.5% of centers.

No adequate standards for protection against infectious risk (22.5%), shortage of personnel to ensure a proper surgical team (12.5%), medico-legal doubts (10.0%), and administrative problems (5.0%) were the other reported obstacles.

### Problems during the post-OR period and actions aimed at improving the management of neonates with surgical conditions

During the post-OR period, the most frequently encountered problems were the excess of fluids administered during surgery, resulting in excessive weight gain after surgery (65.9%); the failure to maintain both adequate body temperature (63.4%) and acid–base balance (41.5%), and tracheal tube malposition and consequent dysventilation (12.2%) (Table 3).

**Table 3:** Problems in OR and actions aimed at improving the management of surgical neonates

In your NICU, the most frequent problem in the post-OR surgery period is: (more than one answer is possible)	*N* = 41/42
Excess fluids administered during surgery, resulting in excessive weight gain after surgery	27/41 (65.9)
Failure to maintain adequate body temperature (e.g., hypothermia/hyperthermia)	26/41 (63.4)
Altered acid–base balance (metabolic acidosis/respiratory alkalosis)	17/41 (41.5)
Orotracheal tube malposition and consequent dysventilation	5/41 (12.2)
Are there anesthesiologists in your center dedicated to managing neonates with surgical conditions?	*N* = 42
Yes	30/42 (71.4)
No	12/42 (28.6)
Are there care protocols in your center for perioperative management of newborn infants with specific surgical conditions? If yes, indicate at least two	N = 42
No	24/42 (57.1)
Yes (Total = 35: OE = 8, CDH = 11, NEC = 7, CPAM = 1, PDA = 6, ROP = 1, TD = 1)	18/42 (42.9)
In your center, to improve the management of newborn infants with surgical pathology, the following are foreseen: (more than one answer is possible)	*N* = 42
Periodic meetings between neonatologists, surgeons, and anesthesiologists for the discussion of complex clinical cases	27/42 (64.3)
Participation of neonatologists, surgeons, and anesthesiologists in study groups dedicated to newborns with surgical pathology	12/42 (28.6)
Conducting clinical studies with the production of scientific publications	7/42 (16.7)
None of the above	12/42 (28.6)

*CPAM* congenital pulmonary airway malformations, *OE* oesophageal atresia, *OR* operating room, *PDA* patent ductus arteriosus, *ROP* retinopathy of prematurity, *TD* thoracic drainage

Among the actions aimed at improving the management of neonates with SC were the presence of anesthesiologists dedicated to the neonate (71.4%) and the use of care protocols for the PM of newborn infants with specific SC (42.9%).

Periodic meetings between neonatologists, surgeons, and anesthesiologists (64.3%), participation in study groups dedicated to newborns with surgical pathology (28.6%), and conducting clinical studies (16.7%) were the other actions aimed at improving surgical neonate management.

### Characterization of bedside surgery concerning the time of experience with BS practice

In relation to the time of experience with BS practice, no significant difference was found in the patient criteria to undergo BS, the surgical pathologies managed at the bedside, and obstacles to the implementation of BS between centers practicing BS for 10 years or less and those practicing for more than 10 years (Table 4).

**Table 4:** Characterization of BS in relation to the time of experience with BS practice

In your center, does bedside surgery facilitate collaboration between neonatologists and anesthesiologists?	*N* = 40	How long has BS been practiced in your NICU?		*p* value (^C^ = chi-square test; ^F^ = Fisher’s exact test)
		< = 10 years (*N* = 14)	> 10 years (*N* = 26)	
Yes	34/40 (85.0)	12 (85.7)	22 (84.6)	1.0^F^
No	6/40 (15.0)			
What are the criteria for BS in your center? (More than one answer is possible)	*N* = 40			
All newborn infants with surgical pathology	1/40 (2.5)	0 (0.0)	1 (3.9)	1.0^F^
Gestational age < 28 weeks and/or birthweight < 1000 g	25/40 (62.5)	7 (50.0)	18 (69.2)	0.23^C^
Infants with clinical instability, regardless of gestational age and birth weight	23/40 (57.5)	7 (50.0)	16 (61.5)	0.48^C^
Depends on ventilatory mode (e.g., neonates on HFOV)	18/40 (45.0)	5 (35.7)	13 (50.0)	0.39^C^
Infants on extracorporeal oxygenation	5/40 (12.5)	2 (14.3)	3 (11.5)	1.0^F^
Other	4/40 (10.0)	3 (21.4)	1 (3.9)	0.12^F^
Which surgical pathology is preferentially managed at the bedside in your center? (more than one answer is possible)	*N* = 40			
Abdominal surgery (NEC, SIP, abdominal wall defects)	27/40 (67.5)	9 (64.3)	18 (69.2)	1.0^F^
Thoracic surgery (CDH, CPAM)	16/40 (40.0)	6 (42.9)	10 (38.5)	0.79^C^
Heart surgery (PDA ligation)	33/40 (82.5)	11 (78.6)	22 (84.6)	0.68^F^
Placement of drainage (pulmonary, abdominal, bladder)	34/40 (85.0)	11 (78.6)	23 (88.5)	0.65^F^
Other	4/40 (10.0)	1 (7.1)	3 (11.5)	1.0^F^
What are the biggest challenges to BS in your center? (up to 3 options)	*N* = 40			
Structural critical issues (absence of adequate physical space)	19/40 (47.5)	9 (64.3)	10 (38.5)	0.12^C^
No adequate standards for protection against infectious risk	9/40 (22.5)	3 (21.4)	6 (23.1)	1.0^F^
Shortage of personnel to ensure an adequate surgical team	5/40 (12.5)	2 (14.3)	3 (11.5)	1.0^F^
Preference of the surgical-anaesthesiology team to perform surgery in standard OR	31/40 (77.5)	9 (64.3)	22 (84.6)	0.23^F^
Administrative problems	2/40 (5.0)	2 (14.3)	0 (0.0)	0.12^F^
Medico-legal doubts	4/40 (10.0)	1 (7.1)	3 (11.5)	1.0^F^

*BS* bedside surgery, *CDH* congenital diaphragmatic hernia, *CPAM* congenital pulmonary airway malformations, *HFOV* high-frequency oscillatory ventilation, *NEC* necrotizing enterocolitis, *NICU* neonatal intensive care unit, *OR* operating room, *PDA* patent ductus arteriosus, *SIP* spontaneous intestinal perforation

## Discussion

The survey results found that, in Italy, the neonatologist was usually involved during the intraoperative management of neonates with SC in 42.9% of the RC. This percentage increased to 50.0% when the surgery was performed at the patient's bedside. The intraoperative involvement means the neonatologist is always near the anesthesiologist for the entire surgical procedure.

It has been reported that the 30-day mortality rate among neonates undergoing anesthesia for surgical and non-surgical procedures ranges between 3.20 and 3.86% [12–14], and it is even higher, reaching almost 11%, among infants with gestational age less than 37 weeks at the time of surgery [9]. Considering the high incidence of perioperative critical events [14], particularly in preterm-born infants, and the reported postoperative mortality, the degree of involvement of the neonatologist that emerges from this survey deserves to be increased. In particular, the management of neonates with SC should require the involvement of a multidisciplinary team in which qualified healthcare professionals, such as a neonatologist and/or a dedicated anesthesiologist, should be the clinical reference figures.

From the results of this survey, we can observe that the involvement of the neonatologist was limited, in most cases (85.7%), to sharing the newborn's clinical issues with the anesthesiologist before the surgery. In contrast, the involvement during the intraoperative phase was less frequent.

It is not surprising that in more than half of the RC (57.1%), the tracheal intubation of the newborn before surgery was performed by the neonatologist. Tracheal intubation is one of the most commonly performed procedures in the NICU, but also one of the most stressful, which also qualifies the neonatologist from other specialists [15]. Additionally, the reported incidence of difficult tracheal intubation among infants admitted to intensive care units was 14%, which is higher than that in pediatric and adult intensive care units [16]: this would explain the tendency to entrust the tracheal intubation of the neonate to the neonatologist before the surgery.

The percentage of Italian centers where BS is practiced was 92.5%, higher than that found by a previous Italian survey, mainly focused on the practice of BS in Italy, which was 70.0% [17]. BS's main criteria were extremely low gestational age, birth weight, and critical clinical conditions. These criteria are consistent with what was reported in the literature about BS in NICUs, usually reserved for those newborns deemed to be too ill or clinically unstable to be transported to a standard OR [18–25].

We found that the main procedures performed at the patient’s bedside were drainage placement, patent ductus arteriosus ligation, and abdominal surgery. The mentioned findings are associated with medical procedures related to clinically unstable or very small neonates, such as the positioning of pleural or abdominal drainage, the ligation of the patent ductus arteriosus, and the laparotomy for necrotizing enterocolitis. These procedures align with the current criteria for BS. Thoracic surgery for congenital diaphragmatic hernia repairs or congenital pulmonary airway malformation corrections is performed at the patient’s bedside in 40.0% of Italian centers where BS is practiced; this is in agreement with the increasing reports about this kind of surgery performed at patients’ bedside [26–28].

An interesting finding of the survey is that the criteria for BS, the type of surgical procedures performed at the patient's bedside, and the barriers to implementation of BS are comparable between centers with 10 years of experience or less and those with more than 10 years of BS experience. Probably, longer experience with BS does not necessarily lead to greater confidence, allowing for more complex bedside surgeries or encountering fewer problems when implementing BS. This could also justify the result of the surgical-anesthesiology team's preference to perform surgery in a standard OR, which in this survey was the major obstacle to BS in 77.5% of centers.

The problems most reported in the post-OR period, such as excess liquids administered during surgery, failure to maintain the body temperature and acid–base balance, and dysventilation due to the poorly positioned tracheal tube, indicate that a high level of skill is required to manage the neonate with SC, to reduce complications potentially linked to surgery.

The main actions aimed at improving the management of neonates with SC were the presence of anesthesiologists dedicated to the neonate and the periodic meetings between neonatologists, surgeons, and anesthesiologists to discuss complex clinical cases. Care protocols for the PM of newborn infants with specific SC are available only in 42.9% of RC. This last data indicates a non-negligible critical issue, not only Italian but international, namely the need to implement care protocols for the management of neonates with SC, as they can reduce the length of hospital stay, surgical complications, and costs of hospitalization [29].

This study has limitations inherent to the nature of the surveys. The lack of responses from 18% of the centers interviewed prevented us from providing a 100% faithful image of the management of neonates with SC in Italy. However, the response rate of 82%, higher than 70%, which is the cut-off point usually viewed with skepticism [11], represents the strength of this study, as it showed that clinicians are interested in the topic. The response rate of this survey allowed us to highlight, with good approximation, the critical issues of the management of neonates with SC that still need to be researched more through multicentric prospective studies in the future.

## Conclusions

This survey illustrates that in Italy, there is still no general consensus regarding the involvement of the neonatologist in the PM of neonates with surgical conditions, and no specific care protocols are widely used. However, due to their unique physiology, newborn infants need both an exclusive care team and specific guidelines for their surgical management. The neonatologist and the dedicated neonatal anesthesiologist should work side by side throughout the surgical procedure with prespecified functions, for example, ventilator management, fluid management, administration of drugs, and analgesics. With the nurses’ invaluable support, this scenario should be more easily realized in the NICU. With this objective, nationally and internationally, standardized PM guidelines, which also consider the adequacy of healthcare professionals, are required to optimize surgical neonates’ care. Moreover, randomized controlled trials are desirable to provide definitive evidence about the safety and efficacy of bedside surgery in improving the care and outcomes of this fragile category of patients.

## Supplementary Information

Below is the link to the electronic supplementary material.

Supplementary file1 (DOCX 21 KB)

## Data Availability

The data that support the findings of this study are available from the corresponding author, SC, upon reasonable request.
