# Supplementary material for: Current management of surgical neonates: is it optimal or do we need to improve? A national survey of the Italian Society of Neonatology
Source: Pediatr Surg Int. 2024 Apr 16;40(1):109. doi: 10.1007/s00383-024-05680-6 (PMC11018645; doi:10.1007/s00383-024-05680-6)
Supplement: Supplementary file 1 — Supplementary file1 (DOCX 21 KB) [file 383_2024_5680_MOESM1_ESM.docx]

**SURVEY ON THE PERIOPERATIVE MANAGEMENT OF PRETERM AND TERM NEONATES WITH SURGICAL CONDITIONS IN ITALY.**

1. In your NICU, is the neonatologist involved in the intra-operative management of newborn infants with surgical conditions?

- Yes, always
- No
- The neonatologist is involved only if the surgery is performed at the bedside in the NICU

1. What is the collaboration between neonatologists and anesthesiologists regarding the management of infants with surgical pathology? (more than one answer is possible)

- All peri- and intraoperative management is referred to the anesthesiologist
- Neonatologist and anesthesiologist share all critical issues before surgery
- The infant is intubated by the neonatologist and then referred to the anesthesiologist
- The neonatologist is involved in the management of ventilation during surgery only in case of high-frequency oscillatory ventilation
- The neonatologist is involved in the management of ventilation during surgery regardless of the ventilation modality
- The neonatologist is involved in fluid management during surgery
- The neonatologist is involved in the pharmacological management of anesthesia
- Neonatologist and anesthesiologist share antibiotic prophylaxis
- Neonatologist and anesthesiologist share post-operative pain management

1. Is bedside surgery performed in your NICU?

- Yes
- No

1. How long has bedside surgery been practiced in your NICU?

- Less than 5 years
- 5-10 years
- More than 10 years

1. does bedside surgery facilitate collaboration between neonatologists and anesthesiologists in your center?

- Yes
- No

1. What are the criteria for bedside surgery in your centre? (more than one answer is possible)

- All newborn infants with surgical pathology
- Gestational age <28 weeks and/or birthweight <1000 grams
- Infants with clinical instability, regardless of gestational age and birth weight
- Depends on ventilatory needs (e.g., neonates on HFOV or requiring inhaled nitric oxide)
- Infants on extracorporeal oxygenation
- Other ________________________________________________

1. Which surgical pathology is preferentially managed at the bedside in your centre? (more than one answer is possible)

- Abdominal surgery (necrotizing enterocolitis, single bowel perforation, abdominal wall defects)
- Thoracic surgery (congenital diaphragmatic hernia, congenital pulmonary malformations)
- Heart surgery (patent ductus arteriosus ligation)
- Placement of drainage (pulmonary, abdominal, bladder)
- Other __________________________________________________

1. What are the biggest challenges to bedside surgery in your center? (Mark up to 3 options)

- Structural critical issues (e.g., absence of adequate physical space)
- No adequate standards for protection against infectious risk
- Shortage of personnel to ensure an adequate surgical team
- Preference of the surgical-anaesthesiology team to perform surgery in standard operating rooms
- Administrative problems
- Medico-legal doubts

1. In your NICU, the most frequent problem in the post-operating room surgery period is: (more than one answer is possible)

- Excess fluids administered during surgery, resulting in excessive weight gain after surgery
- Failure to maintain adequate body temperature (e.g., hypothermia/hyperthermia)
- Altered acid-base balance (metabolic acidosis/respiratory alkalosis)
- Orotracheal tube malposition and consequent dysventilation

1. Are there anesthesiologists in your center dedicated to managing neonates with surgical conditions?

- Yes
- No

1. Are there care protocols in your center for perioperative management of newborn infants with specific surgical conditions? If yes, indicate at least two

- No
- Yes

1.__________________________________________________

2.__________________________________________________

1. In your center, in order to improve the management of newborn infants with surgical pathology, the following are foreseen: (more than one answer is possible)

- Periodic meetings between neonatologists, surgeons, and anesthesiologists for the discussion of complex clinical cases
- Participation of neonatologists, surgeons, and anesthesiologists in study groups dedicated to the newborn with surgical pathology
- Conducting clinical studies with the production of scientific publications
- None of the above
